# A Critical Reassessment of the Kidney Risk Caused by Tetrastarch Products in the Perioperative and Intensive Care Environments

**DOI:** 10.3390/jcm12165262

**Published:** 2023-08-12

**Authors:** Csaba Kopitkó, Tibor Fülöp, Mihály Tapolyai, Tibor Gondos

**Affiliations:** 1 Department of Anesthesiology and Intensive Therapy, Uzsoki Teaching Hospital of Semmelweis University, Uzsoki u. 29–41, H-1145 Budapest, Hungary; 2 Department of Medicine, Division of Nephrology, Medical University of South Carolina, 96 Jonathan Lucas Street, Charleston, SC 29425, USA; 3 Medicine Service, Ralph H. Johnson VA Medical Center, 109 Bee St, Charleston, SC 29401, USA; mtapolyai@aol.com; 4 Szent Margit Hospital, Bécsi út 132, H-1032 Budapest, Hungary; 5 Doctoral School of Pathological Sciences, Semmelweis University, Üllői út 26, H-1088 Budapest, Hungary; gondos.tibor54@gmail.com

**Keywords:** hydroxyethyl starch, acute kidney injury, hemodynamic monitoring, sepsis, cardiac, postoperative

## Abstract

***Purpose:*** To reassess the results of former meta-analyses focusing on the relationship between novel HES preparations (130/0.4 and 130/0.42) and acute kidney injury. Previous meta-analyses are based on studies referring to partially or fully unpublished data or data from abstracts only. ***Methods*:** The studies included in the former meta-analyses were scrutinized by the authors independently. We completed a critical analysis of the literature, including the strengths, weaknesses and modifiers of the studies when assessing products, formulations and outcomes. ***Results*:** Both the published large studies and meta-analyses show significant bias in the context of the deleterious effect of 6% 130/0.4–0.42 HES. Without (1) detailed hemodynamic data, (2) the exclusion of other nephrotoxic events and (3) a properly performed evaluation of the dose–effect relationship, the AKI-inducing property of 6% HES 130/0.4 or 0.42 should not be considered as evidence. The administration of HES is safe and effective if the recommended dose is respected. ***Conclusions*:** Our review suggests that there is questionable evidence for the deteriorating renal effect of these products. Further well-designed, randomized and controlled trials are needed. Additionally, conclusions formulated for resource-rich environments should not be extended to more resource-scarce environments without proper qualifiers provided.

## 1. Introduction

In clinical practice, one of the most common interventions is volume expansion in those with perceived hypovolemia. Intravenous fluid administration is easily performable with crystalloid and colloid infusions or with various blood products. In the current era, the isotonic but non-physiologic 0.9% saline and balanced solutions are available as crystalloid infusions, whereas the 6% hydroxyethyl starch (HES) (130/0.4 or 0.42) and the 5% or 20% human albumin are available as colloids, respectively.

Formerly, dextrans, gelatin and early generations of HES were also available as well. Dextran products are high (40–200 kDa) molecular weight polymers of glucose produced by bacteria (*Leuconostoc mesenteroides*) in sucrose-rich environments [1]. Their volume expansive effect is quite significant. Unfortunately, the risk of life-threatening allergic reactions to dextran products is prohibitively high. While these reactions are preventable by the administration of hapten (1 kDa dextran) a few minutes before the infusion, this property of dextran makes it unsuitable for use in acute situations. An alternative, gelatin infusion was manufactured by partial hydrolysis and chemical modifications after extraction from animal (pig, calf, fish) bones, skin and tendon (molecular weight: 30–35 kDa, concentration: 3–5%) [2]. Their volume-expanding effect is limited and their administration carries the risk of prion-mediated disease transmission. HES preparations are plant-derived products featured at various concentrations (6%, 10%), molecular weights (450 kDa, 200 kDa, 130 kDa) and molar substitutions (0.7, 0.6, 0.5, 0.42, 0.4) [3,4]. This latter property needs some explanation for further interpretation. A molar substitution of 0.7 means that on average there are 7 hydroxyethyl groups for 10 glucose molecules. The evolution of HES generations is as follows: (1) hetastarch–6% HES 450/0.7, (2) hexastarch–6% HES 200/0.6, (3) pentastarch–6%/10% HES 200/0.5 and (4) tetrastarch–6% HES 130/0.4. Other properties such as the C2:C6 hydroxylation ratio, or whether it is made from potato or waxy maize, are generally not labeled on the infusion bottle. The C2:C6 hydroxylation ratio—which potentially affects the elimination of the molecule or its blood coagulation compromising effect—has shown an increasing tendency in commercial products over the years (9:1 in currently available solutions) [5,6]. All dextrans, gelatins and older generation HESs are now removed from the market for various reasons [3,4]. More recently, the use of 6% HES (130/0.4 or 0.42) has been restricted by the European Medicines Agency and the U.S. Food and Drug Administration as its deleterious effects on kidney function came to light [3,7]. On 24 May 2022, the European Commission issued a suspension of the marketing authorizations of HES solutions for infusion in the EU (https://www.ema.europa.eu/en/news/hydroxyethyl-starch-solutions-infusion-recommended-suspension-market (accessed on 11 February 2022, updated on 26 July 2022). The opportunity was given for the individual EU Member States to delay the suspension for no longer than 18 months and keep HES solutions on the market. However, conclusions derived from resource-rich environments, such the EU is, should not be extended to more resource-scarce environments without proper qualifiers. Albumin, the ideal “volume expander”, remains expensive and its supply is ultimately limited. While current methods are safe for preventing the transmission of prion-like illnesses with human albumin preparations, all these are contingent on resource investment and societal wealth to support them [8,9].

Early hemodynamic stabilization can be crucial in the prevention of AKI regarding the short warm ischemic time of the kidneys [10,11]. A promising tool to discriminate between hypovolemic and normovolemic patients is the hypovolemic index (values between 0 and 1) [12]. This parameter is capable of separating these groups of patients (threshold: 0.5), but its validation is still in progress. The first step for hemodynamic stabilization is to achieve euvolemia, which is a wide gray zone without clear boundaries between the volume-sensitive and volume-resistive circulatory states [13,14]. Interstitial accumulation of intravenously administered fluids can increase the renal parenchymal pressure dramatically, and therefore the fluid resuscitation with crystalloids is only a question under debate [15]. The evaluation of kidney perfusion by ultrasound can aid in finding the right balance between fluids and vasoactive drug therapy.

At the same time and over the past several years, the definition of acute kidney failure has become increasingly precise, fostering earlier diagnosis and standardization across the world. The first systematic, universal definition of acute kidney injury (AKI) was accepted in 2002 (RIFLE criteria) and has been followed by three other generally established ones (AKIN, KDIGO, KDIGO with biomarkers) [16,17,18,19]. The studies conducted with third-generation HES show wide differences in the definition of deteriorating renal function, as discussed further below. The severity stages of AKI do not correspond equivocally between the AKI definitions, making it harder to generate a robust comparison [20]. AKI itself has multiple possible causes and is featured by different microhemodynamics and humoral/cellular changes depending on the underlying pathological processes [21]. Two meta-analyses on this topic were performed in 2013, which also included a few studies conducted with the older generation of HES culminating in harmful renal consequences [22,23]. Two other meta-analyses were conducted in recent years to demonstrate the advantages and disadvantages of the administration of 6% 130/0.4–0.42 HES in surgical and trauma patients, proving it safe and favorable in terms of hemodynamic properties [24,25].

It is important, however, to recognize that modern HES products may have value due to their low cost, easy storage and represent a meaningful potential alternative in resource-scare environments. Albumin, although an ideal volume expander, remains expensive and its supply is ultimately limited. While current methods are safe for preventing the transmission of prion-like illnesses with human albumin preparations, all these are contingent on resource investment and societal wealth to support them [8,9]. To further complicate the scenario, we also recognize that the use of plasma expanders may not entirely come from the expansion of plasma volume. A quantity of 250 milliliters of 5% albumin is really 12.5 mL of albumin, which is a syringeful; it is unlikely to only work by expansion of the intravascular space [26]. Shimizu K. et al. have shown in an elegant study that the injection of 20 mL of “plasma expander” hypertonic saline or hypertonic glucose increased blood pressure by suddenly increasing endogenous vasopressin even though plasma volume only increased by 2.3%. The injection of 200 mL of isotonic saline, while expanding plasma volume by 12.7%, did not increase vasopressin levels. 

The aim of our narrative review is to conduct a critical re-assessment of the literature on the safety and efficacy of one specific product, the currently used 6% HES (130/0.4 or 0.42), with regard to renal function, independently of any industrial ties or potential conflicts of interest. Although the indication of tetrastarch is also a clinically relevant point, we have not discussed it due to the limited length of the manuscript. Only safety and efficacy concerns are conferred. 

## 2. The Brief Pathophysiology of AKI

In high-income countries, the three main forms of AKI are the postoperative, the septic and the AKI of cardiac origin, except for forms caused by nephrotoxic agents [21,27]. After noncardiac surgeries, the leading cause of renal dysfunction is the ischemic-reperfusion injury due to general or local hemodynamic instability, transport hypoxia due to blood loss and increased intraabdominal pressure [27]. In cardiology patients, venous congestion and with on-pump cardiac surgery, the activation of the immune system is added to these confounders as a significant contributing factor [28]. Hypovolemia and congestive cardiac insufficiency are accompanied by the high activity of the renin-angiotensin-aldosterone system (RAAS) in contrast to the low activity of the RAAS due to hypervolemia, resulting in absolutely different renal microcirculation. Given the presence of renal capsules in in situ kidneys, with venous congestion, fluid overload and third-spacing, the interstitial pressure can exponentially rise within the kidney parenchyma [29]. It is to be understood that from an evolutionary biological standpoint, one would expect fewer escape mechanisms to evolve for surviving fluid overload than coping with hypovolemia. However, septic AKI is characterized by a different intrarenal hemodynamics: the dilatation of the efferent glomerular arteries and the increased patency of shunt vessels produce a low-pressure-high-flow state, consequently dropping the filtration rate in the glomeruli [30]. Besides circulatory changes, several inflammatory mediators play a crucial role in the progression of septic AKI. However, the main contributor of AKI is hemodynamic instability with a potential contribution of nephrotoxic agents, as described recently [31].

## 3. The Diagnostic Uncertainties of AKI

The worsening of kidney function represents a continuum. Since no clear boundaries can be observed between physiological and pathological conditions, it is difficult to define infliction points. Despite several known pitfalls, most generally accepted diagnostic systems employ the rise of serum creatinine and the amount of urine output as the basis for detecting AKI [16,17,18,19]. However, serum creatinine concentration is considered a ‘slow-reacting parameter’: serum creatinine levels follow clinical changes with an outstanding delay. Moreover, the definition of the perceived “baseline” serum creatinine level further qualifies the perceived frequency and severity of AKI [32]. Using eGFR (as suggested by the Acute Dialysis Quality Initiative [ADQI]) or minimum inpatient serum creatinine levels as the baseline inflated the incidence of AKI in comparison to the most recent outpatient serum creatinine levels between 7 and 365 days prior to admission (38.3%, 35.9% vs. 25.5%, *p* < 0.001, respectively) [33]. However, the first admission serum creatinine level underestimated the incidence of AKI compared to the most recent outpatient serum creatinine concentration (13.7% vs. 25.5%, *p* < 0.001) [33]. In this study, the main differences (both false positive and negative) were in the AKIN 1 stage. Based on data from the Beginning and Ending Supportive Therapy for the Kidney (BEST Kidney) study, the estimated serum creatinine (Modification of Diet in Renal Disease [MDRD]) leads to a bidirectional misclassification of patients at enrollment (false negative for Risk: 7.3%; false positive for Failure: 18.7%, false positive for all AKI: 11.7%) and at admission to ICU (false positive for Injury, Failure, all AKI: 5.5%, 14%, 18.8%, respectively) [34]. Muscle wasting, sarcopenia, racial differences and fluid overload are important qualifier to interpret serum creatinine values in the ICU settings [32]. In an attempt to overcome these difficulties, newer markers (e.g., cystatin C, NGAL, TIMP2 × IGFBP7) are implemented, but their general usefulness is debated [21,27]. Urine output is an important parameter contributing to the diagnostic frequency and severity of AKI, but administering diuretics blurs the diagnostic reliability [35].

## 4. Studies Conducted with 6% HES 130/0.4 or 0.42 Analyzing Its Renal Effects

The designs of studies conducted to evaluate the deleterious renal effects of 6% HES 130/0.4 or 0.42 (the different molar substitution value represents products of different manufacturers) are listed in Table 1. Four large meta-analyses were published aiming to evaluate the relationship between the administration of HES and the development of AKI [22,23,24,25]. Two investigators (CsK, TG) scrutinized all the studies included in the systematic reviews independently. The outcomes and the investigators’ critical remarks can be found in Table 2. Two trials (Safety and Efficacy of a 6% Hydroxyethyl Starch Solution vs. an Electrolyte Solution in Trauma Patients (TETHYS); Safety and Efficacy of 6% Hydroxyethyl Starch Solution vs. an Electrolyte Solution in Patients Undergoing Elective Abdominal Surgery (PHOENICS)) are officially registered (NCT03338218, NCT03278548), but no results have been published to date [36,37].

One of the meta-analyses published in 2013 was based on 10 studies concerning RRT with extremely low reported heterogeneity (τ^2^ = 0, I^2^ = 0%) [22]. The largest included study (CHEST, weight: 51.8%; *n* = 6651) was conducted in hypovolemic patients at any time in the ICU, but the percentage of septic patients was about 23–25% in both groups [38,39]. They found a significantly lower incidence of AKI at either the Risk or Injury stage and a non-significant difference in the Failure stage. The only finding referring to kidney damage was the slightly higher rate (7.0% vs. 5.8%, *p* = 0.04) of RRT in the HES group, but the initiation of RRT was based on the clinicians’ discretion; objective criteria were not communicated. The authors’ opinion was that this did not affect the study results since the clinicians were unaware of study-group assignment. The second larger study (6S, weight: 20.8%; *n* = 798) was conducted in septic patients and the diagnostic criteria of AKI were different from the generally accepted systems [40]. Nevertheless, the relative risk of AKI was similar in the intervention and the control groups. It is to be noted that patients with AKI at the time of randomization were included with equal frequency in the two groups. There was another study conducted on septic patients (CRYSTMAS, weight: 3.9%; *n* = 196) which showed no significant difference in the incidence of AKI [41]. Similar courses of serum creatinine and biomarkers were observed in both study groups. One small study from China included in the research was designed to demonstrate the effect of HES on intraabdominal pressure [42]. The diagnosis of AKI was based on urine output. No data were reported about renal replacement therapy in this study. Another small study contained no data on renal function [43]. One of the included studies was in abstract form; four others were conducted with the second generation of HES (the sum of the weight of these five studies was altogether 22.7%) [44,45,46,47]. 

Generally speaking, hemodynamic data and nephrotoxic agents are reported only in a few studies. Interestingly, the recommended dose of tetrastarch was exceeded in many studies, but this point has been underemphasized in the systematic reviews to date, and none of them investigated a dose–side-effect relationship between the 6% HES 130/0.4 or 0.42 and AKI. Another critical aspect is the possibility of overcorrection of hypovolemia. To date, several methods have been described and employed to detect volume status, but none of them can guide the fluid therapy precisely [48]. Despite the fact that the role of fluid overload in developing AKI is well known, it is not mentioned in any study conducted with tetrastarch.

In the Cochrane library, a systematic review was performed to analyze the effects of HES on kidney function [23]. This review is from studies referring partially [38,44,45,49,50,51,52,53,54] or entirely [43,55,56] to unpublished data or data from abstracts only [57]. Certain studies included in the research are from published data only [40,41,46,47,58,59,60,61,62,63,64,65,66,67,68] and one of them contains no data on renal function [43]. The high (≥200 kDa) and lower than nowadays commercially available (70 kDa) molecular weight HES solutions are also included. Renal outcomes were determined according to the RIFLE criteria, need of RRT or by the authors’ definition. We included in our analysis all the studies conducted with 6% 130/0.4–0.42 HES; the details can be found in Table 1 and Table 2. The included trials were not selected based on patients’ subgroups. Only a statement made in the main text of the article indicates that non-septic patients had fewer adverse effects, but the divergent types of HES make it hard to draw a relevant conclusion for everyday practice in 2023. This systemic review was helpful at the time of writing, but several new data have emerged since then.

One recent meta-analysis (heterogeneity for both AKI and RRT: τ, I^2^ = 0%) reported that a 6% 130/0.4 HES is safe against different comparator fluids in various subgroups of patients [24]. The authors included three studies for demonstrating AKI in cardiac and eleven trials in non-cardiac/mixed surgery patients. One of the cardiac surgery [69] and one of the non-cardiac surgery [70] trials were designed as noninferiority studies, and two other cardiac surgery [71,72] and ten non-cardiac surgery [70,73,74,75,76,77,78,79,80,81] trials were observational. One of the cardiac trials [62] (weight: 0.5%, total weight of cardiac studies: 6.8%) and two of the non-cardiac ten [76,82] did not report the renal function appropriately (weight: N/A); one applied a 24 h follow-up [75] only, while another one compared HES derived from maize and from potato (weight: 17.5%) [83]. The sample size of these studies is less than 100 patients, with two exceptions [73,78] (*n* = 386 and 534). Both cardiological and non-cardiological surgery studies consider HES to be at least non-inferior regarding renal safety parameters for crystalloids, gelatin and 5% human albumin.

The planning process of the studies has several methodological problems, which can exert a significant impact on the results. A good example is the second largest study (CRISTAL) conducted on septic patients [84]. In this trial, crystalloids were administered to only one fourth of the patients in the colloid group. In everyday practice, the first intravenous fluid administered is a crystalloid of any kind (0.9% saline, balanced or hypotonic solution), and colloids are considered second-line drugs [85]. In certain studies, a proportion of patients received HES before randomization, an aspect that remained unanalyzed [40,41,86]. Hemodynamic instability itself can lead to impaired kidney function and may be an ongoing issue in sepsis or postoperative states [87,88]. Surprisingly, hemodynamic data (e.g., the duration and severity of the hypoperfusion period, any organ-specific cessation of renal blood flow during surgery, ultrasonographic data about intrarenal blood flow and venous congestion, etc.) were not reported in most of the studies (Table 2). The results are confusing from the perspective of renal detrimental effects as well. The implemented definition of deteriorated kidney function varies in a wide range between decreasing urine output and fulfilling KDIGO criteria with biomarkers. Finally, nephrotoxic mediators and agents are very common in sepsis patients and during intensive care therapy. However, these factors or the lack of these factors are usually not indicated.

We must also consider discriminating among the trials according to different patient subpopulations because of the previously mentioned distinct patho-mechanisms of AKI. The largest studies (over 1000 patients per group) were conducted only in septic patients, while some middle-sized (500–1000 patients per group) studies were steered in patients who had undergone abdominal surgery and only small studies are available in cardiac surgery patients (Table 1). In multi-center studies, a significant heterogeneity can be observed among data produced by different centers, but the results are not provided according to investigator sites. A further shortcoming is the missing logistic regression analysis. If we assume that HES is an independent influencing factor in the development of kidney failure, it is then critical to be verified by a multivariate logistic regression analysis. In the studies where the harmful impact of HES on kidney function was referred to, no such analysis was carried out in any but one of these; this particular study failed to identify any relationship between the worsening of kidney function (AKI) and the administration of tetrastarch [89]. A major shortcoming of all the studies assuming the kidney-damaging effect of HES is that none of them performed a dedicated dose–side-effect analysis. If a drug is harmful, it can be rightly assumed that side-effects occur more often with higher doses and longer use. Based on the data, there would have been an opportunity for this in many investigations, but in no case was such an analysis carried out. As a consequence of this methodological deficiency, in the opinion of the authors of this review, any current meta-analysis is inevitably distorted.

Studies performed in cardiac patients do provide more hemodynamic data but show no significant difference in AKI or the need for RRT [50,52,62,69,90,91]. Studies conducted on postoperative patients after abdominal surgery compared HES with other colloids [63,64,70,83,92], while others did so against crystalloids [73,74,78] and some against both [53]. These studies proved that HES is not inferior to other colloids or crystalloid infusions. Even in cases where the risk of AKI seems to be higher in the HES group, the 95% CI saddles on 1.0, indicating that relative risk is uncertain.

**Table 1:** Designs of studies conducted with 6% HES 130/0.4 or 0.42.

Study	Trial Design/Country/ Type of Patients	Study Fluids	Indication and Dose (Planned, Maximal and Cumulative) of HES	Endpoints	Definition of Renal Endpoint
**Septic patients**					
**Perner,** **2009–2011,** **published in 2012 (6S)** [40]	Prospective, multi-center, parallel, blinded, randomized Denmark, Norway, Finland and Iceland Severe sepsis, septic shock	6% (130/0.42) HES–398 patients Ringer’s acetate–400 patients	**Indication**: volumen expansion **Planned**: 33 mL/kg daily **Daily maximal**: 50 mL/kg (exceeded only in case of two patients) **Cumulative**: 44 mL/kg (IQR: 24–75 mL/kg) *(~3168 mL/patients)*	**Primary:** composite (death or dependence on dialysis) at 90 days **Secondary:** ○ the development of AKI ○ percentages of days alive without RRT	Use of RRT, or A renal SOFA score ≥ 3, or Plasma creatinine level > 179 μmol/L or urinary output < 500 mL/d Doubling of the plasma creatinine level
**Müller,** **2015** [86]	Post-hoc analysis of 6S trial [9] Denmark, Norway, Finland and Iceland Severe sepsis, septic shock	6% (130/0.42) HES–398 patients Ringer’s acetate–400 patients	**Indication**: volumen expansion **Planned**: 33 mL/kg daily **Daily maximal**: 50 mL/kg) (exceeded only in case of two patients) **Cumulative**: 44 mL/kg (IQR: 24–75 mL/kg) *(~3168 mL/patients)*	Daily average AKI stage Trajectories of the AKI stages Hazard risk of increasing or decreasing AKI stage Time to initiation of RRT Intervention effect on mortality for AKI Effect of increasing AKI stage on mortality	KDIGO Missing baseline creatinine values were estimated using MDRD formula
**Dubin, 2010** [93]	Prospective, bi-center, randomized, controlled, pilot trial Two centers in Argentina Severe sepsis	6% (130/0.4) HES–9 patients 0.9% saline–11 patients	**Indication**: intravenous volume expansion to increase microvascular flow index (MFI) **Planned**: unknown **Daily maximal**: unknown **Cumulative**: unknown	Sublingual microcirculatory parameters Fluid balance	Creatinine (baseline and at 24 h) Urine output
**Guidet, 2012** **(CRYSTMAS)** [41]	Prospective, multi-center, double-blind, randomized, active-controlled 24 centers in France and Germany Severe sepsis	6% (130/0.42) HES–100 patients 0.9% saline–96 patients	**Indication**: (initial) hemodynamic stabilization **Planned**: unknown Fluid intake prior randomization: 35.5 ± 25.3 mL/kg) **Daily maximal**: 50 mL × kg^−1^ × d^−1^ on the first day; 25 mL × kg^−1^ × d^−1^ from the second to the fourth day **Cumulative**: 1379 ± 886 mL, 2615 ± 1499 mL over four consecutive days	**Primary:** the amount of study drug required to achieve initial hemodynamic stabilization at the end of first four hours **Secondary:** ○ time taken to achieve initial hemodynamic stabilization, ○ total quantity of study drug infused over four consecutive days in the intensive care unit	RIFLE AKIN ARF: a two-fold increase in serum creatinine from baseline or need for renal replacement therapy NAG NGAL α1-microglobulin
**Myburgh, 2012** **(CHEST)** [38,39]	Prospective, multi-center, parallel, blinded, randomized, controlled 32 centers in Australia and New Zealand Hypovolemic patients at any time in the ICU	6% (130/0.42) HES–3315 patients 0.9% saline–3336 patients	**Indication**: correction of hypovolemia **Planned**: unknown **Daily maximal**: unknown. Daily dose: 526 ± 425 mL *(~6.6 ± 5.3 mL/kg)* **Cumulative**: unknown	**Primary:** all-cause mortality at 90 days, **Secondary:** ○ incidence of AKI ○ the use of RRT ○ new organ failures for cardiovascular, respiratory, coagulation and liver systems	RIFLE
**Annane, 2013 (CRISTAL)** [84]	Prospective, multi-center, parallel, randomized 57 centers in France, Belgium, Canada, Algeria and Tunisia Sepsis, multiple trauma, hypovolemic shock	Crystalloid infusions–1443 patients (isotonic saline, hypertonic saline, buffered solutions) Colloid–1414 patients (hypooncotic (eg. gelatines, 4% or 5% of albumin), hyperoncotic (eg. dextrans, hydroxy-ethyl starches and 20% or 25% of albumin)	**Indication**: fluid resuscitation **Planned**: unknown **Daily maximal**: 30 mL/kg **Cumulative**: 1500 mL (95% CI: 1000–2000 mL), *(*~*21.4 mL/kg [14.3–28.6 mL/kg])* 973 patients (68.8%), duration 2 (95% CI: 1–2) days	**Primary:** mortality at 28 days **Secondary:** ○ death rates at 90 days and at ICU and hospital discharge, ○ number of days alive and not receiving RRT, mechanical ventilation or vasopressor therapy ○ days without organ system failure (i.e., SOFA score < 6), days not in the ICU or hospital for 28 days from ICU admission	Need of renal replacement therapy (indications were not presented)
**Cardiac surgery patients**					
**Gallandat** **2000** [50]	Prospective, multi-center, parallel, randomized, double-blind, clinical, phase III study Two centers in the Netherlands Coronary artery bypass surgery	6% (130/0.42) HES in saline–30 patients 6% (200/0.5) HES–29 patients	**Indication**: acute normovolemic hemodilution + priming the heart-lung machine + intra/postoperative fluid management **Planned**: 500 mL for hemodilution, 1000 mL for priming the heart-lung machine **Daily maximal**: 3000 mL *(*~*36.1 mL/kg)* **Cumulative**: intraoperatively: 1475 ± 100 mL *(~17.8 mL/kg)*, postoperatively: 1150 ± 511 mL *(~13.9 mL/kg)*, total: 2550 ± 561 mL (31.0 ± 7.4 mL/kg) in 130/0.4 HES group	**Primary:** compare the total volume of colloids (HES plus isotonic pasteurized plasma) infused per treatment group from induction of anesthesia until 16 h after the end of surgery **Secondary:** ○ hemodynamics, ○ blood gases, ○ fluid balance	Urine output Serum creatinine
**Van der** **Linden,** **2005** [52]	Prospective, single-center, single-blind, randomized, open controlled noninferiority study regarding ○ hemodynamics ○ fluid balance ○ coagulation parameters ○ serum creatinine ○ liver enzymes Belgium Coronary artery bypass surgery	6% (130/0.4) HES–64 patients modified fluid gelatine–68 patients	**Indication**: priming the heart-lung machine + postoperative fluid management **Planned**: not reported **Daily maximal**: 50 mL × kg^−1^ × d^−1^ **Cumulative**: 21.3 ± 8.3 mL/kg (~1683 ± 656 mL) intraoperatively, 27.5 ± 12.6 mL/kg *(~2173 ± 995 mL)* postoperatively, 48.9 ± 17.2 mL/kg *(~3863 ± 1359 mL)* total	Hemodynamic data Fluid balance Laboratory data	Serum creatinine Urine production
**Ooi, 2009** [72]	Prospective, single-center, single-blind, randomized, controlled Malaysia Coronary artery bypass surgery	6% (130/0.4) HES–45 patients succinylated gelatine–45 patients	**Indication**: priming the heart-lung machine + intra/postoperative fluid management **Planned**: not reported **Daily maximal**: 50 mL × kg^−1^ × d^−1^ **Cumulative**: intraoperatively: 1225.6 ± 158.3 mL *(*~*17.5 mL/kg)*, first 24 h postoperatively: 716.7 ± 910.2 mL *(*~*10.2 mL/kg)*, total: 1942.3 ± 1046.1 mL (27.7 mL/kg) in HES group	**Primary:** postoperative blood loss **Secondary:** ○ transfusion of blood products ○ total volume of colloids infused per treatment group intraoperatively and in the first 24h postoperatively ○ renal function ○ complications related to colloid usage	eGFR based on MDRD formula
**Skhirtladze, 2014** [94]	Prospective, single-center, randomized, controlled, double-blind trial Austria Elective cardiovascular surgery [i.e., CABG, valve repair or replacement and surgery of the ascending aorta] on cardiopulmonary bypass	HA group: 5% albumin up to 50 mL × kg^−1^ × day^−1^–76 patients HES group: 6% HES 130/0.4 up to 50 mL × kg^−1^ × day^−1^–81 patients RL group: RL up to 50 mL × kg^−1^ × day^−1^–79 patients	**Indication**: priming the heart-lung machine + intra/postoperative fluid management **Planned**: 1500 mL for priming, intraoperative dose was restricted to 33 mL × kg^−1^ × d^−1^ **Daily maximal**: 50 mL × kg^−1^ × d^−1^ **Cumulative**: intraoperatively: 2500 (IQR: 2250–2750) mL, postoperatively: 625 (IQR: 50–1000) mL, total: 3000 (IQR: 2750–3500) mL in HES group	**Primary:** clinical bleeding based on chest tube drainage over the first 24 h after cardiopulmonary bypass **Secondary:** ○ serum creatinine ○ transfusion of PRBCs and other blood products ○ changes in hemoglobin and hemostatic parameters	Renal dysfunction defined as serum creatinine 1.5 mg/dL Delta creatinine (maximal creatinine value within 48 h minus baseline creatinine)
**Joosten, 2016** [77]	Prospective, single-center, parallel, double-blinded, randomized, controlled Belgium Elective cardiovascular surgery on cardiopulmonary bypass (CPB)	6% (130/0.4) maize HES–59 patients 6% (130/0.42) potato HES–59 patients	**Indication**: priming the heart-lung machine + intra/postoperative fluid management **Planned**: 1000 mL for priming *(*~*13 mL/kg)*, intraoperative dose in 250 mL boluses to maintain SVV <13% **Daily maximal**: 50 mL × kg^−1^ × d^−1^ **Cumulative**: intraoperatively: 1000 mL (IQR: 000–1250 mL) *(*~*13 [IQR: 13–16 mL/kg])* in maize and 1000 mL (IQR: 1000–1200 mL) *(~13 [IQR: 13–16 mL/kg])* in potato HES (NS); up to POD2: 1950 mL (IQR: 1250–2325 mL) *(~25 [IQR: 16–29 mL/kg])* mL in maize HES and 2000 mL (IQR: 1500–2700 mL) *(~27 [IQR: 20–66 mL/kg])* mL in potato HES (NS)	**Primary:** Calculated blood loss up to POD2 **Secondary:** ○ short and long-term effects of study fluids on postoperative renal function	Short-term: AKIN and requirement of RRT Long-term: urea, creatinine, eGFR (CKD-EPI formula)
**Svendsen,** **2018** [91]	Prospective, single-center, randomized, controlled study, blinded for all participating investigators except for the perfusionist Norway Coronary artery bypass surgery	6% (130/0.42) HES–20 patients Ringer’s acetate–20 patients	**Indication**: priming the heart-lung machine **Planned**: 1700 mL for priming **Daily maximal**: unknown **Cumulative**: unknown	Fluid balance Hemoglobin, hematocrit, platelets, coagulation parameters (TEG)	AKIN
**Duncan, 2020** [69]	Prospective, single-center, randomized, controlled, triple-blind, parallel-group, non-inferiority study USA Scheduled aortic valve replacement	6% (130/0.42) HES–69 patients 5% human albumin–72 patients	**Indication**: hypovolemia **Planned**: 250 or 500 mL boluses if hypovolemia detected by monitoring of cardiac index, HR, systolic blood pressure, vasopressor requirement and CVP/PCWP or in case of severe acute surgical haemorrhage **Daily maximal**: 35 mL × kg^−1^ × day^−1^ **Cumulative**: unknown	**Primary:** urinary NGAL at baseline, 1 h after arrival to ICU and 24 h after completion of surgery **Secondary:** ○ changes in hemostatic parameters ○ urinary IL-18 ○ all-cause one-year mortality ○ kidney function at 6 and 12 months	RIFLE and see also Endpoints
**Postoperative patients after abdominal surgery**					
**Mahmood** **2007** [63]	Prospective, single-center, randomized Denmark, Norway, Finland and Iceland Severe sepsis, septic shock	6% 200/0.62 HES–21 patients 6% 130/0.4 HES–21 patients 4% gelatine–20 patients	**Indication**: maintenance infusion during and after the surgery **Planned**: 3 mL/kg bolus of colloid followed by a maintenance rate of 2 mL × kg^−1^ × h^−1^ during surgery and increased to maintain a urine output greater than 0.5 mL × kg^−1^ × h^−1^. Further colloid administration was based on maintenance of MAP over 85 mmHg and CVP between 8 and 10 cmH_2_O **Daily maximal**: 3911 ± 1783 mL *(*~*51* ± *23 mL/kg)* in 130/0.4 HES group **Cumulative**: from 8 h before surgery to 24 h after the surgery: 3443 ± 1769 mL *(~45 ± 23 mL/kg)* in 200/0.62 HES group 3911 ± 1783 mL *(~51 ± 23 mL/kg)* in 130/0.4 HES group	**Serum creatinine** **BUN** **Urinary IgG:creatinine ratio** **α1-microglobulin:creatinine ratio**	See Endpoints
**Godet, 2008** [70]	Prospective, multi-center, parallel, open, randomized, controlled Seven centers in France Postoperative patients after abdominal aortic surgery	6% (130/0.42) HES in saline–29 patients 3% modified fluid gelatine–31 patients	**Indication**: maintenance infusion during and after the surgery **Planned**: according to anesthesiologist’s judgement during surgery based on MAP, CVP, fluid balance and the need of catecholamines **Daily maximal**: 50 mL × kg^−1^ × d^−1^ **Cumulative**: Day 1: 1709 ± 836 mL (23.9 ± 11.9 mL/kg) Day 2: 1577 ± 714 mL (21.8 ± 9.5 mL/kg) Day 3: 1780 ± 752 mL (24.8 ± 10.5 mL/kg) Day 4: 1862 ± 1171 mL (25.4 ± 15.4 mL/kg) Day 5: 1874 ± 1308 mL (26.2 ± 17.7 mL/kg) Day 6: 1779 ± 1204 mL (24.0 ± 16.2 mL/kg) Total (day 1– day 6): 10 237 ± 4561 mL (139.7 ± 58.2 mL/kg)	**Primary renal safety parameter:** the peak increase in serum creatinine up to POD6 or hospital discharge **Secondary:** ○ renal dysfunction defined as serum creatinine above the upper limit of normal plus an increase of ≥44.2 mmol/L (≥0.5 mg/dL) above baseline at any time point after the end of surgery ○ the minimum postoperative CrCl ○ the incidence of oliguria (urine output < 500 mL/day) ○ urinary NAG	CrCl: ○ mild (≥50 mL/min) ○ moderate (30–50 mL/min) ○ severe (<30 mL/min)
**Mukhtar** **2009** [64]	Prospective, single-center, randomized Egypt Patients scheduled for living donor liver transplantation	6% 130/0.4 HES–20 patients 5% albumin–20 patients	**Indication**: maintenance infusion during and after the surgery **Planned**: 250 mL bolus based on maintenance of CVP and/or PAOP between 5 and 7 cmH_2_O **Daily maximal**: 50 mL × kg^−1^ × d^−1^ during the intraoperative period and first 4 postoperative days **Cumulative**: intraoperatively: 3080 ± 417 mL, postoperatively: 6229 ± 1140 mL in 130/0.4 HES group	AKI Duration of postoperative mechanical ventilation Start of enteral feeding Pulmonary complications	Creatinine clearance Cystatin C
**Yang 2011** [53]	Prospective, single-center, randomized China Hepatectomy	6% (130/0.4) HES–30 patients 20% human-albumin–30 patients Ringer’s lactate–30 patients	**Indication**: maintenance infusion during and after the surgery **Planned**: 1000 mL/d *(*~*16 mL/kg)* in POD1–3 and 500 mL/d *(*~*8 mL/kg)* on POD4–5 **Daily maximal**: unknown **Cumulative**: intraoperatively: 3484.6 ± 1072.5 mL *(~56* ± *17 mL/kg)*, total: 10,235.0 ± 393.9 mL *(~165 ± 6 mL/kg)* in 130/0.4 HES group	Child–Turcotte–Pugh grading MELD score C-reactive protein, IL-6 Pulmonary complications Nosocomial infections Bleeding In-hospital mortality	BUN Creatinine
**Demir, 2015** [92]	Prospective, multi-center, randomized Turkey Living-donor liver transplantation	6% (130/0.4) HES–18 patients 4% gelatine–18 patients	**Indication**: maintenance infusion during the surgery **Planned**: according to hemodynamic data (SVV, CVP, MAP) **Daily maximal**: unknown **Cumulative**: 2.3 ± 0.8 L *(*~*32* ± *11 mL/kg)* in 130/0.4 HES group	Renal endpoints	BUN, Creatinine (eGFR: Cockroft-Gault, MDRD, CKD-EPI)
**Ghodraty, 2017** [74]	Prospective, bi-center, parallel, double-blinded, randomized, controlled Iran, USA Small intestine resection	6% (130/0.4) HES–46 patients Ringer’s lactate–45 patients	**Indication**: maintenance infusion during the surgery **Planned**: 2 mL × kg^−1^ × h^−1^ as a maintenance fluid plus fluid loss in 1:1 ratio **Daily maximal**: unknown **Cumulative**: 10.4 ± 4.1 mL/kg	**Primary:** the time of the first flatus or bowel movement **Secondary:** ○ AKI ○ surgical complications	AKIN
**Joosten, 2018** [83]	Prospective, bi-center, parallel, double-blinded, randomized, controlled, superiority trial Two centers in Belgium Scheduled open abdominal surgery (patients required unexpected suprarenal aortic crossclamping were excluded)	6% (130/0.4) waxy maize HES in balanced crystalloids–80 patients balanced crystalloids–80 patients	**Indication**: maintenance infusion during the surgery **Planned**: EGDT (multiple 100-mL mini-fluid challenges) based on hemodynamic measurements (SVV; closed-loop system) **Daily maximal**: 33 mL/kg **Cumulative**: 900 mL (IQR: 400–1300 mL) *(*~*13 mL/kg [IQR: 6–18 mL/kg])* intraoperatively. Only one patient (1%) reached the maximal dose	**Primary:** POMS score at POD2 **Secondary:** ○ the effect of study fluids on postoperative renal function ○ cardiac, pulmonary, gastrointestinal, renal, infectious complications ○ coagulation ○ surgical complications up to 30 days after surgery	KDIGO Requirement of RRT
**Kammerer, 2018** [95]	Prospective, single-center, parallel, single-blinded Germany Scheduled for cystectomy	6% (130/0.4) HES–47 patients 5% human-albumin–53 patients	**Indication**: replacement of blood loss in 1:1 ratio during the surgery, postoperative fluid management **Planned**: replacement of blood loss in 1:1 ratio during the surgery, postoperative fluid management **Daily maximal**: 30 mL/kg **Cumulative**: 2000 ± 969 mL *(~27 ± 13 mL/kg)*	**Primary:** serum cystatin C ratio between POD 90 and preoperative values **Secondary:** ○ eGFR ○ NGAL ○ RIFLE on POD 3 and POD 90 ○ change of serum cystatin C levels ○ need for vasopressors and catecholamines up to POD 3	Serum cystatin C RIFLE
**Werner, 2018** [89]	Prospective, multi-center, parallel, double-blinded, randomized Three tertiery care centers Germany	balanced 10% HES 130/0.42–20 patients balanced 6% HES 130/0.42–22 patients balanced crystalloid–21 patients	**Indication**: intraoperative fluid management **Planned**: EGDT (multiple 100-mL mini-fluid challenges) based on hemodynamic measurements (SVV) **Daily maximal**: 30 mL/kg for 10% HES; 50 mL/kg for 6% HES **Cumulative**: 2250 (IQR: 1750–3000 mL); 33.3 mL/kg (IQR: 28.2–46.2 mL/kg for 6% HES)	**Primary:** the intraoperative volume of HES **Secondary:** ○ AKI ○ fluid balances ○ hemodynamics	KDIGO (as post-hoc analysis)
**Kabon,** **2019** [78]	Prospective, multi-center, parallel, double-blinded, randomized One center in Austria, two centers in USA Postoperative patients after major abdominal surgery (open or laparoscopically assisted)	6% HES 130/0.4 in 0.9% saline–523 patients Ringer’s lactate–534 patients	**Indication**: intraoperative volume replacement **Planned**: 250 mL over 5 min based on esophageal Doppler measurements (stroke volume, corrected aortic flow time) **Daily maximal**: 1500 mL **Cumulative**: 1 (IQR: 0.5–1.5) liter	**Primary:** a composite of major complications (cardiac, pulmonary, infectious, gastrointestinal, renal, coagulation) **Secondary:** a composite of minor complications the primary composite augmented by readmission and mortality **Safety:** ○ in-hospital serum creatinine concentrations ○ serum creatinine concentration up to 6 months postoperatively	Maximum postoperative serum creatinine concentration (stages 1–3 of AKI are not clearly defined)
**Futier, 2020** **(FLASH)** [73]	Prospective, multi-center, double-blind, parallel, randomized 20 university hospitals in France Postoperative patients after major abdominal surgery	6% HES 130/0.4 in 0.9% saline–389 patients 0.9% saline–386 patients	**Indication**: intraoperative volume replacement **Planned**: 250 mL over 5 min to maximize stroke volume; in case of less than a 10% increase in stroke volume, the study fluid administration was stopped **Daily maximal**: 30 mL × kg^−1^ × d^−1^ (100 patients [10.5%] of patients received more) **Cumulative**: intraoperatively: 1000 mL (IQR: 750–1500 mL) (~12 mL/kg [IQR: 9–18 mL/kg]); postoperatively: 500 mL (IQR: 500–750 mL) *(*~*6 mL/kg [IQR: 6–9 mL/kg])*; POD2: 500 mL (IQR: 250–1000 mL) *(~6 mL/kg [IQR: 3–14 mL/kg])*; total: 33.4 ± 3.4 mL/kg in HES group *(~2739 ± 279 mL)*	**Primary:** a composite of mortality or at least one of the following by POD14: AKI, pulmonary, cardiovascular, infectious or surgical complication **Secondary:** ○ major postoperative complications to POD14. ○ kidney dysfunction: oliguria (24-h urine output < 500 mL), KDIGO score ○ major adverse cardiovascular events ○ pulmonary complications ○ SIRS score on POD2 ○ SOFA score without GCS on POD2 ○ time to return of bowel function (flatus and stool)	KDIGO
**Others**					
**Neff 2003** [65]	Prospective, single-center, randomized, controlled Switzerland Craniocerebral trauma	6% (130/0.42) HES–16 patients 6% (200/0.5) HES + 5% albumin–15 patients	**Indication**: volume replacement in the ICU for up to 28 days **Planned**: repetitive large doses **Daily maximal**: 70 mL × kg^−1^ × d^−1^ **Cumulative**: 2297 ± 610 mL *(*~*30* ± *8 mL/kg)* daily; total: 19 ± 16 L *(~246 ± 208 mL/kg)* (max: 66 L!) 20 mL × kg^−1^ × day^−1^: *n* = 16, mean duration: 4.8 days 30 mL × kg^−1^ × day^−1^: *n* = 16, mean duration: 3.9 days 40 mL × kg^−1^ × day^−1^: *n* = 13, mean duration: 3.1 days 50 mL × kg^−1^ × day^−1^: *n* = 12, mean duration: 2.0 days 60 mL × kg^−1^ × day^−1^: *n* = 10, mean duration: 1.8 days 70 mL × kg^−1^ × day^−1^: *n* = 3, mean duration: 1.0 day	Safety of HES 6% (130/0.4) with regard to coagulation and renal function	Not specified
**James, 2011** **(FIRST)** [58]	Prospective, single-center, double-blind, randomized, controlled USA Penetrating and blunt trauma	6% (130/0.42) HES–36 patients with penetrating, 20 patients with blunt trauma 0.9% saline–31 patients with penetrating, 22 patients with blunt trauma	**Indication**: fluid resuscitation **Planned**: undetermined **Daily maximal**: 33 mL × kg^−1^ × d^−1^ **Cumulative**: Penetrating trauma: 5093 ± 2733 mL *(~70 ± 38 mL/kg)*; Blunt trauma: 6113 ± 1919 mL *(~79 ± 25 mL/kg)*	**Primary:** volume of resuscitation fluid in the first 24 h, tolerance of full enteral feeding by POD5 **Secondary:** ○ use of blood product ○ biochemical abnormalities, particularly lactate, chloride, and acid–base and hemostatic disturbances ○ SOFA scores **Safety:** ○ AKI	RIFLE
**Tyagi 2019** [80]	Prospective, single-center, double-blind, randomized, controlled India Scheduled orthopedic surgery under general anesthesia with >200–300 mL blood loss expected	6% (130/0.42) HES–19 patients Ringer’s lactate–19 patients	**Indication**: intraoperative fluid replacement **Planned**: If SVV was >10% in supine or lateral position, or >14% in prone position, a bolus of 100 mL of the intervention fluid was infused over 2–4 min **Daily maximal**: not applicable **Cumulative**: 689 ± 394 mL *(*~*12* ± *7 mL/kg)*	AKI NGAL Urine output The volume of intervention fluid Blood loss	KDIGO NGAL

Abbreviations: AKI: Acute Kidney Injury; AKIN: Acute Kidney Injury Network; ARF: Acute Renal Failure; BUN: Blood Urea Nitrogen; CI: Confidential Interval; CPB: Cardiopulmonary Bypass; CrCl: Creatinine Clearance; CVP: Central Venous Pressure; EGDT: Early Goal Directed Therapy; eGFR: estimated Glomerular Filtration Rate; GCS: Glasgow Coma Scale; HA: Human Albumin; HES: Hydroxyethyl Starch; ICU: Intensive Care Unit; IgG: Immunglobulin G; IQR: Interquartile Range; KDIGO: Kidney Disease: Improving Global Outcome; MAP: Mean Arterial Pressure; MDRD: Modification of Diet in Renal Disease; MELD: Model of End-Stage Liver Disease; NAG: β-N-Acetyl-β-D-Glucosaminidase; NGAL: Neutrophil Gelatinase-Associated Lipocalin; PAOP: Pulmonary Arterial Occlusion Pressure; POD: Postoperative Day; POMS: Profile of Mood States; RIFLE: Risk, Injury, Failure, Loss, End-stage renal disease criteria for acute kidney injury; RL: Ringer’s Lactate; RRT: Renal Replacement Therapy; SIRS: Systemic Inflammatory Response Syndrome; SOFA: Sepsis-related Organ Failure Assessment; SVV: Stroke Volume Variation.

**Table 2:** Endpoints, outcomes and criticism of studies conducted with 6% HES 130/0.4 or 0.42.

Study	Main Outcomes	Authors Conclusion	Additional Information	Does the Study Definitely Support That in Respect of Kidney Function the HES Is	
				Detrimental	Safe
**Septic patients**					
**Perner,** **2009–2011,** **published in 2012 (6S)** [40]	90-day mortality: 51% in HES, 43% in Ringer’s acetate group (RR: 1.17 [95% CI: 1.01–1.36], *p* = 0.03) AKI: 41% in HES, 35% in Ringer’s acetate group (RR: 1.18 [95% CI: 0.98–1.43], *p* = 0.08) RRT: 22% in HES, 16% in Ringer’s acetate group (RR: 1.35 [95% CI: 1.01–1.08], *p* = 0.04)	Patients with severe sepsis who received fluid resuscitation with HES 130/0.42, as compared with those who received Ringer’s acetate, had a higher risk of death at 90 days, were more likely to receive RRT	The authors did not assess all cointerventions during the trial period, “because the trial was large, was blinded, and used stratified randomization, it is less likely that any imbalance in concomitant interventions affected the results.” 52% of all patients received colloids (<1000 mL) before randomization The indications of RRT were not communicated **Hemodynamic status**: unknown **Nephrotoxic drugs**: similar number in both groups **Logistic regression analysis**: not performed **Dose-effect relationship**: not investigated	**No**	**No**
**Müller,** **2015** [86]	The incidence of AKI was higher in the HES group (28% vs. 22%; *p* = 0.04) at 5 days The average AKI stage was 0.2 higher in the HES group at 5 days (*p* < 0.01) An increase in AKI stage by one was associated with increased mortality (hazard risk: 1.35; 95% CI, 1.22–1.49; *p* < 0.01) The trajectories, the incidence of AKI, the hazard of increase or decrease in AKI stage were not different at 90 days When adjusted, the interventions’ effects on mortality for AKI were not different at 90 days The fraction of patients on RRT was higher in the HES group (17% vs. 12%, *p* = 0.03)	The occurrence of AKI and the initiation and use of RRT beyond day 5 did not differ between the two intervention groups The excess 90-day mortality caused by HES may, at least in part, have been mediated through AKI Patients with AKI at baseline were included the use of RRT was not protocolized	The authors did not assess all cointerventions during the trial period Patients with acute kidney injury at the time of randomization were included with equal frequency in the two intervention groups The differences experienced at 5 days mostly became insignificant at 90 days Shorter periods on RRT are more frequent in the HES group The indication of RRT is uncertain in ¼–⅕ of -patients in both groups The results are not discriminated according to the molecular size of HES **Hemodynamic status**: unknown **Nephrotoxic drugs**: unknown **Logistic regression**: not performed **Dose-effect relationship**: not investigated	**No**	**Partly yes**
**Dubin, 2010** [93]	Normal serum creatinine levels in both groups (on admission: 1.2 ± 0.3 vs. 2.1 ± 1.2 mg/dL; at 24 h: 1.5 ± 0.5 vs. 2.3 ± 1.6 mg/dL) Similar urine output in both groups (1825 ± 863 mL vs. 1507 ± 1350 mL, NS)	Pilot study A better recruitment of the microcirculation in tetrastarch group	**Hemodynamic status**: unknown **Nephrotoxic drugs**: unknown **Logistic regression**: not performed	**No**	**Yes**
**Guidet, 2012** **(CRYSTMAS)** [41]	28-day mortality: 31.0% in HES and 25.3% saline group (NS) 90-day mortality: 40.0% in HES and 34.0% saline group (NS)ARF: 24.5% in HES and 20.0% saline group (NS)–comparable according to both RIFLE and AKIN classifications Course of mean serum creatinine: similar in both groups (the highest values were 155 ± 109 in HES and 152 ± 106 μmol/L in saline group Urinary biomarkers: similar course in both groups	6% HES 130/0.4, had no negative effects on mortality, kidney function, coagulation, or pruritus	**Hemodynamic status**: shorter time (11.8 ± 10.1 vs. 14.3 ± 11.1 h; NS) and less study fluid (1379 ± 886 mL vs. 1709 ± 1164; *p* = 0.0185) to initial hemodynamic stabilization in the HES group; no significant difference in cathecolamines dose **Nephrotoxic drugs**: unknown	**No**	**Yes**
**Myburgh, 2012** **(CHEST)** [38,39]	AKI: risk: 54.0% in the HES group, 57.3% in the saline group (*p* = 0.007); RR: 0.94 (95% CI: 0.90–0.98) Injury: 34.6% in the HES group, 38.0% in the saline group (*p* = 0.005); RR: 0.91 (95% CI: 0.85–0.97) Failure: 10.4% in the HES group, 9.2% in the saline group (NS); RR: 1.12 (95% CI: 0.97–1.30) RRT: 7.0% in the HES group, 5.8% in the saline group (*p* = 0.04); RR: 1.21 (95% CI: 1.00–1.45)	21% relative increase in the number RRT in the HES group RRT was initiated at the discretion of the attending clinicians HES was associated with increased urine output in patients with less severe AKI Serum creatinine levels were consistently higher in the HES group, suggesting a progressive reduction in creatinine clearance and more severe AKI	The baseline creatinine was not provided The criteria for the initiation of RRT were not outlined in the protocol The diagnosis of AKI was significantly based on urinary output (R: 52.7% of 54.0% and 56.5% of 57.3%, I: 36.2% of 34.6% and 39.7% of 38% (!), F: 11.6% of 10.4% and 10.5% of 9.2% (!) in the HES and the saline group, respectively) Neither the dose of diuretics nor the information about diuretic-naïvity of the patients were provided The RR remained lower in the Risk and Injury category in the HES group after adjustments The RR of Failure category and of RRT, the 95% CI saddles on 1.00 making its relevancy dubious A possibly misleading figure can be found in the main text plotting the serum creatinine within the first 6 days in the ICU (no SD values, no daily comparison of the levels, not clearly defined how the *p*-values were calculated) Neither the daily, nor the cumulative dose of HES were published. As it can be gleaned from the chart in the supplementary material of the source paper, 1000 mL was administered in the first, 500 mL in the second day and minimal after that time. SD-s were not plotted Mixed critically ill population, neither the basic SOFA scores, nor the proportion of different subpopulations were provided The logistic regression of contributors for RRT is missing Several organ systems are listed among the 90-day cause specific mortality, but renal causes are missing **Hemodynamic status**: unknown **Nephrotoxic drugs**: unknown, but more transfusions in HES group **Logistic regression**: not performed	**No**	**No**
**Annane, 2013 (CRISTAL)** [84]	No increase in risk of renal replacement therapy and in risk of death	No evidence for a colloids-related increase in the risk for renal replacement therapy	Open labeled fluids The study was powered to compare crystalloid vs. colloid strategies Long inclusion time (2003–2012): the definition of AKI and several therapeutic guidelines have been changed during this period The type of HES is not specified (older generations of HES were available in the study period) and the study fluids in the colloid group are highly heterogenous The septic subpopulation (54.7% in colloid, 54.0% in crystalloid group) was not evaluated thoroughly 86% of patients in the crystalloids group received a chloride-rich solution (ie, isotonic saline) 70% of patients in the colloids group received HES and 35% of them received gelatins **Hemodynamic status**: unknown **Nephrotoxic drugs**: unknown	**No**	**No**
**Cardiac surgery patients**					
**Gallandat** **2000** [50]	No difference in HR, MAP, CVP, PCWP and cardiac index between groups There were no significant differences in urine output between the groups (3635 ± 1015 mL vs. 3581 ± 941 mL, NS) in 130/0.4 and 200/0.5 HES group, respectively There were no significant differences between the groups in serum creatinine measured on POD1 (84.1 ± 15.7 µmol/L vs. 83.9 ± 15.5 µmol/L, NS) and POD2 (108.5 ± 17.3 µmol/L vs. 94.0 ± 20.6 µmol/L, NS) in 130/0.4 and 200/0.5 HES group, respectively	The new generation hydroxyethyl starch HES 130/0.4 6% is an effective plasma volume expander compared to the standard HES 200/0.5 6% (pentastarch) in heart surgery	The aim of the study was to demonstrate the noninferiority of low molecular weight HES on the hemodynamics **Hemodynamic status**: all parameters were in the target range ○ cardiac index preferably > 2 L × min^−1^ × m^−2^, ○ filling pressures: CVP 4–12 mmHg and PCWP 6–12 mmHg, ○ urine output of 1–2 mL × kg^−1^ × h^−1^ **Nephrotoxic drugs**: unknown	**No**	**Yes**
**Van der** **Linden,** **2005** [52]	All parameters were comparable between groups Baseline creatinine level: 92.8 ± 20.3 µmol/L At 20 h after the ICU admission: 88.4 ± 23.0 µmol/L POD5: 90.2 ± 25.6 µmol/L Urine output: intraoperatively: 7.4 ± 4.7 mL, postoperatively: 30.0 ± 12.0 mL, total: 37.5 ± 14.0 mL	6% HES 130/0.4 up to 50 mL/kg is a valuable alternative to modified fluid gelatin for plasma volume expansion during and after cardiac surgery	**Hemodynamic status**: No significant difference between groups in preoperative levels and until POD 1 of HR, MAP, MPAP, PAOP, RAP, cardiac index, SI, SVR, SvO_2_ **Nephrotoxic drugs**: unknown	**No**	**Yes**
**Ooi, 2009** [72]	No significant difference in renal outcomes (eGFR preoperatively: 85.3 ± 15.3 mL × min^−1^ × 1.73m^−2^) ○ POD1: 84.1 ± 24.7 mL × min^−1^ × 1.73 m^−2^, ○ POD2: 69.4 ± 21.0 mL × min^−1^ × 1.73 m^−2^, ○ POD4: 80.1 ± 21.4 mL × min^−1^ × 1.73 m^−2^, ○ after 4 weeks: 90.8 ± 21.3 mL × min^−1^ × 1.73 m^−2^ in HES group) No significant difference in other outcome parameters	6% HES 130/0.4 is a safe alternative colloid for priming the CPB circuit and volume substitution in patients undergoing CABG	**Hemodynamic status**: unknown **Nephrotoxic drugs**: unknown	**No**	**Yes**
**Skhirtladze, 2014** [94]	Delta creatinine: −1.8 (−4.4–9.7) μmol/L in HES group RRT: *n* = 2 (2.6%) in HA group; *n* = 1 (1.2%) in HES group; *n* = 0 (0%) in RL group Use of vasopressors: **high dose** (not quantified): 11% in HA group, 21% in HES group, 16% in RL group, **low dose**: 59% in HA group, 54% in HES group, 56% in RL group	The study was not powered to detect differences in major complications (e.g., re-exploration, renal replacement therapy) and mortality	**Hemodynamic status**: unknown **Nephrotoxic drugs**: unknown	**No**	**Yes**
**Joosten, 2016** [77]	No significant difference in outcome parameters	AKI (Stage 1 to stage 3 at POD2) in 17 patients (14%) Need for RRT during the entire ICU length of stay in three patients (2.5%)–consistent with the literature (15–30% for AKI and 2–9% for RRT)	**Hemodynamic status**: unknown **Nephrotoxic drugs**: unknown	**No**	**Yes**
**Svendsen,** **2018** [91]	The cardiac index was higher in the HES group at arrival to the ICU (2.7 ± 0.4 L × min^−1^ × m^−2^ vs. 2.1 ± 0.3 L × min^−1^ × m^−2^; *p* < 0.001) No statistical differences in serum creatinine levels between groups (data just plotted) Three patients in the HES group reached AKI stage 1 postoperatively (NS), but all regained preoperative values within 5–10 days	HES contributed to a 40% reduction in the perioperative fluid balance Better cardiac performance in HES group Power calculations were not performed with respect to this issue before the study	**Hemodynamic status**: No significant difference between groups in preoperative levels and until POD 1 of HR, MAP, CVP, cardiac index, SVRI, ITBVI, EVLWI, GEDVI with the exception of higher cardiac index at ICU admission (see outcomes) **Nephrotoxic drugs**: unknown	**No**	**Yes**
**Duncan, 2020** [69]	Similar results were observed in all measured parameters of kidney function in both groups (Risk: 35 patients–51%, Injury: 6 patients–9%, Failure: 1 patient–1%; at one year: Risk: 1 patient–4%, no Injury, no Failure in the HES group) In the early postoperative phase less patients in the Risk stage in the HES group (51% vs. 67%)	HES would be considered non-inferior if the postoperative urinary NGAL concentrations higher than expected variability did not permit to conclude that HES was non-inferior to albumin The observed long-term kidney outcomes and mortality were similar between groups	**Hemodynamic status**: unknown **Nephrotoxic drugs**: unknown	**No**	**Yes**
**Postoperative patients after abdominal surgery**					
**Mahmood** **2007** [63]	The mean serum creatinine was significantly lower in HES 130/0.4 group than the gelatine group at days 1, 2 and 5 (only plotted, between 80–100 μmol/L at all timepoints) Urinary α1-microglobulin levels were significantly lower in HES 130/0.4 group than in gelatine group at clamp on and then between 4 and 24 h (and at days 4 and 5) Urinary IgG:creatinine ratios were significantly lower in HES 130/0.4 group than in gelatine group at 8 h and day 5 Serum creatinin plotted and seems to continuously decrease from the first to the last time-point (exact values and significance not given) in case of HES 130/0.4 but not with the other colloids	In aortic surgery, when HES infusion is accompanied by approximately twice its volume of crystalloid, there is improved renal function compared with gelatine An appropriately powered study is needed	Total fluid input from 8h before surgery to 24 h after the surgery: 11,770 (IQR: 9880–14,353) mL; PRBC: 6.0 (IQR: 4.0–8.0); balance: 6834.0 (IQR: 5012.5–8544.5) mL **Hemodynamic status**: No significant difference in vasopressor requirement s among groups **Nephrotoxic drugs**: unknown	**No**	**Yes**
**Godet, 2008** [70]	Serum creatinine increased by 23.6 ± 55.3 μmol/L in HES group Day 1: 108.4 ± 29 μmol/L Day 2: 116.0 ± 43.7 μmol/L Day 3: 123.3 ± 61.5 μmol/L Day 4: 118.4 ± 75 μmol/L Day 5: 117.6 ± 69.6 μmol/L Day 6: 113.3 ± 64.2 μmol/L The one-sided 95% CI of [−∞; 21.26 mmol/L] exceeded the defined clinically relevant non-inferiority level of 0.2 mg/dL even after exclusion of two extreme outliers	The choice of the colloid—either HES 130/0.4 (6%) or gelatin—has no impact on renal safety parameters and outcome in patients with decreased renal function undergoing elective abdominal aortic surgery	Use of furosemide postoperatively was discouraged but permitted **Hemodynamic status**: MAP: no statistical difference between the groups (mean MAP > 73 mmHg at any time) **Nephrotoxic drugs**: unknown	**No**	**Yes**
**Mukhtar** **2009** [64]	A minimal transient deterioration was observed in both groups (only plotted)–maximum serum creatinine < 130 μmol/L, which returned to preoperative level o no significant difference in the endpoints greater net cumulative fluid balance in the HES group (3047 ± 2000 mL vs. 1100 ± 900 mL, *p* = 0.029) The other hemodynamic parameters (HR, MAP, CO) were similar	No impact on renal function or patient outcome	**Hemodynamic status**: Goals (CVP: 5–7 mmHg, MAP > 70 mmHg, SVR > 600 dyne × s^−1^ ×cm^−5^, cardiac index > 2.5–3.0 L × min^−1^ × m^−2^) were achieved **Nephrotoxic drugs**: unknown	**No**	**Yes**
**Yang 2011** [53]	The serum levels of creatinine in HES group preoperative: 77.8 ± 20.0 μmol/L, POD1: 73.4 ± 21.6 μmol/L, POD3: 66.9 ± 19.2 μmol/L, POD5: 64.4 ± 18.3 μmol/L) and BUN in HES group preoperative: 5.9 ± 1.7 mmol/L, POD1: 4.6 ± 1.3 mmol/L, POD3: 4.4 ± 1.7 mmol/L, POD5: 4.1 ± 1.3 mmol/L Morbidity and mortality during the study period were not significantly different between HA and HES group, but both were better, then RL group There were no significant differences in intraoperative fluid administration (around 3000–3500 ± 1000 mL) among groups	Equivalent hemodynamics, liver function and postoperative clinical outcomes in HA and HES groups HES may exert more favorable effects on the acute phase response	The study was powered to evaluate the effects of fluid administrating strategy on hepatic function **Hemodynamic status**: Goals (CVP: 5–9 mmHg, MAP: 60–80 mmHg) were achieved **Nephrotoxic drugs**: unknown	**No**	**Yes**
**Demir, 2015** [92]	BUN, raw and Cockroft–Gault-based eGFR were similar between groups, but MDRD and CKD-EPI-based were lower in gelatine group	The use of HES did not cause any renal dysfunction None of the patients AKI advancing to Stage 2 was observed	There is no information about postoperative dose of HES The mentioned formulas are not for detecting AKI **Hemodynamic status**: unknown **Nephrotoxic drugs**: unknown	**No**	**Uncertain**
**Ghodraty, 2017** [74]	Duration of ileus was shorter in HES group (73.4 ± 20.8 h vs. 86.7 ± 23.7 h, *p* = 0.006) No difference in postoperative AKI and anastomotic leak	Colloid fluids may have a preventive role in gastrointestinal operations regarding reduction of postoperative ileus	The study was not powered for estimating AKI The mean body weight calculated from given data is extremely low (41.7 kg) **Hemodynamic status**: unknown **Nephrotoxic drugs**: unknown **Logistic regression**: conducted for determining the contributors of postoperative ileus	**No**	**With significant limitations**
**Joosten, 2018** [83]	Both incidence of complications and POMS score were lower in the colloid group. There was no difference in renal outcomes (KDIGO 1: 13 of 80 patients–16%; KDIGO 2: 6 of 80 patients–8%; KDIGO 3: 1 of 80 patients–1% in HES group) Fewer rescue fluids and vasoactive drugs in colloid group	Colloid-based goal-directed fluid therapy was associated with fewer postoperative complications than a crystalloid one	**Hemodynamic status**: significantly lower SVV in the colloid group intraoperatively (8% [95% CI: 7–9%] vs. 10% [95% CI: 8–13%]), significantly but clinically irrelevantly higher MAP (79 mmHg [95% CI: 74–84 mmHg] vs. 75 mmHg [95% CI: 72–81 mmHg]) and lower HR (67/min [95% CI: 60–76/min] vs. 72/min [95% CI: 64–82/min]) **Nephrotoxic drugs**: unknown	**No**	**Yes**
**Kammerer, 2018** [95]	There were no significant differences between groups in renal function parameters, blood loss and transfusion needs Serum cystatin C in HES group: preoperative: 1.02 (0.83–1.36) mg/L, after surgery: 0.78 (0.65–1.11) mg/L; POD1: 0.94 (0.75–1.14) mg/L, POD3: 0.82 (0.69–1.06) mg/L, POD90: 1.13 (0.94–1.39) mg/L) serum NGAL in HES group: preoperative: 183.6 (136.2–241.8) ng/mL after surgery: 207.4 (152.8–301.8) ng/mL 2–4 h postoperative: 207.2 (156–307) ng/mL POD1: 230.8 (162.2–304) ng/mL POD3: 176.9 (121.4–247.7) ng/mL AKI on POD3: no AKI in HES group; two patients (1%) in Risk, two patients (1%) in Injury phase in albumin group AKI on POD90: five patients (11.6%) in Risk, zero patients in Injury phase in HES group; five patients (11.9%) in Risk, two patients (4.8%) in Injury phase in albumin group	Perioperative 5% albumin and balanced 6% HES solutions have comparable safety profiles with respect to renal function in these patients	**Hemodynamic status**: no differences in intraoperative cardiac output and SVV; no differences in intra- and postoperative vasoactive medication requirements **Nephrotoxic drugs**: unknown	**No**	**Yes**
**Werner, 2018** [89]	No significant differences in the intraoperative volume of HES and the net fluid balance between the colloid groups AKI: ○ creatinine criterion: 46.7% vs. 23.5% vs. 20% in 10% HES vs. 6% HES vs. crystalloid groups, respectively (NS) ○ urine output criterion: 86.7% vs. 58.8% vs. 45% in 10% HES vs. 6% HES vs. crystalloid groups, respectively (10% HES vs. crystalloid: *p* = 0.010, 6% HES vs. crystalloid: NS) ○ after adjustment regarding preventive diuretic administration: 10% HES vs. crystalloid: 86.7% vs. 55.0%, *p* = 0.033, 6% HES vs. crystalloid: 58.8% vs. 55%, NS) ○ combined criteria: 64.7% vs. 65% vs. 86.7% in 10% HES vs. 6% HES vs. crystalloid groups, respectively (NS) Using grey zone approach the lower cut-off of 6% HES: 2000 mL (18.8 mL/kg); the upper cut-off: 2750 mL (45.0 mL/kg) for not experience AKI with near certainty	Although 6% HES might be safe, they recommend that it should be used with caution during surgery and not applied beyond 18.8 mL/kg during surgery	Creatinine and urine output was followed until POD3 No data about postoperative period **Hemodynamic status**: no difference in MAP, CVP and norepinephrine requirements, but significantly higher SVI in 6% HES group **Nephrotoxic drugs**: unknown	**No**	**No**
**Kabon,** **2019** [78]	There were no creatinine differences between the groups over the initial 14 postoperative days (maximum: 73.4 [62.8–88.4] μmol/L) or initial 6 months (maximum: 76.9 [64.5–93.7] μmol/L). AKI did not differ significantly	Doppler-guided intraoperative HES administration did not reduce the number of serious complications HES did not reduce the duration of hospitalization, but there was also no indication of renal toxicity Colloids do not reduce perioperative complications; they should be used in surgical patients because its higher cost	**Hemodynamic status**: no difference in intraoperative TWA MAP and phenylephrine requirements **Nephrotoxic drugs**: unknown	**No**	**Yes**
**Futier, 2020** **(FLASH)** [73]	Significantly higher SVI, lower dose of norepinephrine, higher urine output and higher need for transfusion in HES group There was no significant difference in major post-operative complication rates between the two groups RR: ○ AKI (adjusted): 1.27 (95% CI: 0.96–1.70, *p* = 0.10) ○ o KDIGO stage 1 (adjusted): 1.45 (95% CI: 1.15–1.83, *p* = 0.002) ○ KDIGO stage 2 or 3 (adjusted): 0.98 (95% CI: 0.57–1.68, *p* = 0.95) ○ Kidney function on day 14 (adjusted): 1.27 (95% CI: 0.96–1.70, *p* = 0.1) ○ Need for RRT (adjusted): 0.55 (95% CI: 0.22–1.37, *p* = 0.2) ○ AKI up to day 28 (adjusted): 1.30 (0.98–1.74, *p* = 0.07)	HES was better than crystalloids at expanding intravascular volume Suggested an increased risk of acute kidney injury in association with use of HES The study may not be powered enough to detect a significant difference among subgroups These findings do not support the use of HES for volume replacement therapy in such patients	The trial protocol restricted the use of study fluid to the day of surgery and the next 24 h; administration of fluid later in the hospital course was not controlled All co-interventions undertaken during the study period were not assessed The study population did not include patients with lower risk of morbidity Patients with AKI risk index class 3–5 were included Sepsis developed in 20% of patients up to day 28 100 patients received study fluid at higher doses than the protocol-specified maximum daily dose The use of 0.9% saline rather than a balanced crystalloid solution may have affected the results Most confidential intervals saddle on 1.000 leaving open the possibility of no effect of study fluid on the certain outcomes **Hemodynamic status**: no significant difference intraoperatively in baseline MAP and SVI, the baseline SVI SVI measured at the end of the surgery was higher and the intraoperative dose of norepinephrine was lower in the HES group **Nephrotoxic drugs**: unknown	**No**	**No**
**Others**					
**Neff 2003** [65]	In two patients with multiorgan failure (both in the HES 200/0.5 + 5% albumin group, but not related to colloids The remaining 29 patient: worst creatinine clearance: 93 ± 15 mL/min	Originally planned sample size of 40 patients After 31 subjects had been enrolled and randomized, the institutional ethics committee raised questions regarding the occurrence of intracranial bleeding complications in both groups and requested an interim analysis. The study was not continued after the interim analysis because of safety concerns	The severe neurologic deficit was independent of the assignment of patients into either group **Hemodynamic status**: Goals (MAP ≥ 80 mmHg, cerebral perfusion pressure ≥ 70 mmHg) were achieved, limitations (PAOP ≥ 16 mmHg, CVP ≥ 20 mmHg, signs of cardiac failure) were not exceeded **Nephrotoxic drugs**: unknown	**No**	**Yes**
**James, 2011** **(FIRST)** [58]	HES group: ○ Penetrating trauma: Risk: one patient (3%) Injury: zero patients (0%) RRT: zero patients (0%) ○ Blunt trauma: Risk: seven patients (35%) Injury: four patients (20%) RRT: two patients (8%)	No serious risk of renal injury associated with the use of HES 130/0.4 in acute resuscitation	Serum lactate was lower in the HES group comparing to the control group in case of penetrating, but not in case of blunt trauma **Hemodynamic status**: unknown **Nephrotoxic drugs**: unknown	**No**	**With limitations**
**Tyagi 2019** [80]	Postoperative urinary NGAL **>50 ng/mL**: RL group: 32%, HES group 21% (NS) **>25 ng/mL**: RL group: 47%, HES group: 32% (NS) **Early postoperative AKI**: RL group: 26%, HES group: 21% (NS)	SVV-guided tetrastarch administration may be preferred to Ringer’s lactate in patients undergoing major orthopedic surgery under general anesthesia, due to the significantly better intravenous expansion efficacy, higher cardiac index and an insignificant trend toward better postoperative renal function	**Hemodynamic status**: No significant differences in heart rate, CVP, systolic and diastolic blood pressures **Nephrotoxic drugs**: unknown	**No**	**No**

Abbreviations: *AKI*: Acute Kidney Injury; *AKIN*: Acute Kidney Injury Network; *ARF*: Acute Renal Failure; *BUN*: Blood Urea Nitrogen; *CI*: Confidential Interval; *CO*: Cardiac Output; *CPB*: Cardiopulmonary Bypass; *CVP*: Central Venous Pressure; *EGDT*: Early Goal Directed Therapy; *eGFR*: estimated Glomerular Filtration Rate; *EVLWI*: Extravascular Lung Water Index; *GCS*: Glasgow Coma Scale; *GEDVI*: Global End-Diastolic Volume Index; *HA*: Human Albumin; *HES*: Hydroxyethyl Starch; *HR*: Heart Rate; *ICU*: Intensive Care Unit; *IgG*: Immunglobulin G; *IQR*: Interquartile Range; *ITBVI*: Intrathoracic Blood Volume Index; *KDIGO*: Kidney Disease: Improving Global Outcome; *MAP*: Mean Arterial Pressure; *MDRD*: Modification of Diet in Renal Disease; *MPAP*: Mean Pulmonary Artery Pressure; *NGAL*: Neutrophil Gelatinase-Associated Lipocalin; *NS*: Non-significant; *PAOP*: Pulmonary Arterial Occlusion Pressure; *PCWP*: Pulmonary Capillary Wedge Pressure; *POD*: Postoperative Day; *PRBC*: Packed Red Blood Cell; *RAP*: Right Arterial Pressure; *RIFLE*: Risk, Injury, Failure, Loss, End-stage renal disease criteria for acute kidney injury; *RL*: Ringer’s Lactate; *RR*: Relative Risk; *RRT*: Renal Replacement Therapy; *SD*: Standard Deviation; *SI*: Stroke Index; *SOFA*: Sepsis-related Organ Failure Assessment; *SVI*: Stroke Volume Index; *SvO_2_*: Mixed Venous Oxygen Saturation; *SVR*: Systemic Vascular Resistance; *SVRI*: Systemic Vascular Resistance Index; *SVV*: Stroke Volume Variation; *TEG*: Thromboelastography; *TWA*: Time-Weighted Average.

## 5. Studies Supporting the Beneficial Hemodynamic Effects of HES

Although it was not always their primary endpoint, several of the mentioned studies reported the favorable hemodynamic effects of HES relative to crystalloids [41,42,53,73,82,91] or other colloids, [52,69,95], while a few studies are against the favorable circulatory effects of HES compared to crystalloids [64,94].

A large multi-center controlled randomized study conducted by Gondos et al. found that 6% HES 130/0.4 is a valuable alternative to other colloids [96]; 200 mixed postoperative ICU patients were investigated in this multi-center study. After the baseline hemodynamic evaluation was carried out, 10 mL/kg of lactated Ringer’s solution, succinylated gelatin 4% *w/v*, 130/0.4 hydroxyethyl starch 6% *w/v* (HES) or human albumin 5% *w/v* was administered over 30 min. Hemodynamic measurements were performed at 30, 45, 60, 90 and 120 min. Their findings were supported by Toyoda et al. [97]. These studies clearly showed that both tetrastarch and albumin have significant hemodynamic effects even at 120 min, while the hemodynamic effect of crystalloids disappears within 20 min. Another controlled randomized single-center study conducted in 57 severe sepsis patients compared the hemodynamic effects of 6% (130/0.42) HES (250 mL every 6 h) and 20% human albumin (100 mL every 12 h) [56]. The administration of a crystalloid solution was allowed as it was considered necessary. The hemodynamic goals were MAP > 65 mmHg, intrathoracic blood volume index (ITBVI) > 850 mL × m^−2^ and cardiac index > 3.5 L × min^−1^ × m^−2^. The most common source of sepsis was ventilator-associated pneumonia. The decrease of the alveolar-arterial oxygen gradient (AaDO_2_) was significantly better in the HES group in the first 72 h, with no significant differences in hemodynamic indices. Renal effects were not investigated.

## 6. The Role of Hyperchloremia in the Development of AKI

A substantial bias and debate have emerged about whether we should differentiate the solutions based on their chloride content and how this effect further modifies potential interactions with source colloid materials and the plant they are derived from. One should keep in mind that isotonic saline can lead to both hyperchloremia and a significant increase in total body sodium content. Only one liter of 0.9% NaCl contains three times the recommended daily sodium intake. The entire topic is not discussed here in detail for reasons of limited space, but we cite the study conducted on twelve healthy adult male volunteers [98]. Renal artery blood flow velocity and renal cortical perfusion were compared by magnetic resonance imaging at 0, 30, 60, 120, 180 and 240 min after starting a 30-min intravenous administration of one liter 6% 130/0.4 maize-derived HES in 0.9% NaCl and 6% 130/0.4 potato-derived HES in a balanced solution. The authors found similar mean peak serum chloride levels, blood volume, strong ion difference, serum creatinine to serum NGAL ratios and mean renal artery flow velocities between groups, albeit renal cortical perfusion was significantly increased (7% from the baseline) after the infusion of potato-derived HES in a balanced solution, compared with a 2.5% decrease from the baseline in the case of maize-derived HES in 0.9% saline. The authors reported significant hyperchloremia (109 mmol/L vs. 104 mmol/L, *p* < 0.0001), a greater expansion of extracellular fluid (1484 mL vs. 1155 mL, *p* = 0.029) and the deterioration of both renal artery blood flow velocity (a 13% decline from the baseline, *p* = 0.045) and renal cortical perfusion (an 11.7% reduction from the baseline, *p* = 0.008) after the infusion of two liters of 0.9% NaCl compared with a balanced solution (raised renal circulatory parameters) by the same method [99]. At the end of the four-hour observational period, 14% and 12% of saline and balanced solutions remained in the intravascular compartment, respectively.

## 7. Conclusions

Summarizing these results, it is the opinion of the authors that the administration of HES is safe and effective if the recommended dose is respected. Restoring circulating plasma volume is essential to prevent renal hypoperfusion. Crystalloid solutions alone fill the extravascular and interstitial space, whereas colloids retain a longer intravascular effect duration. The tissue deposition of HES can be minimized by adherence to the manufacturer’s proposal.

Some renal benefits can be achieved by potato-derived HES in a balanced solution. To date, both the published large studies and the meta-analyses show significant bias in the context of the deleterious effect of 6% 130/0.4–0.42 HES. The 6% HES (130/0.4 or 0.42) can have a better hemodynamic profile than crystalloid infusions used alone, but its deleterious effect on kidney function remains questionable. Without (1) detailed hemodynamic data, (2) the exclusion of other nephrotoxic events and (3) a properly performed evaluation of the dose–effect relationship, the AKI-inducing property of the 6% HES 130/0.4 or 0.42 could not be accounted for as evidence. We need some well-designed randomized controlled trials to appropriately explore and reflect on clinical problems.

## Data Availability

A preliminary version of this manuscript has been posted via Preprints.org 2023, 2023060168. https://doi.org/10.20944/preprints202306.0168.v1, with on-line posting date of 2 June 2023.

## Abbreviations

**AaDO_2_**	Alveolar-Arterial Oxygen Gradient
**AKI**	Acute Kidney Injury
**AKIN**	Acute Kidney Injury Network
**ARF**	Acute Renal Failure
**BUN**	Blood Urea Nitrogen
**CABG**	Coronary Artery Bypass Grafting
**CI**	Confidential Interval
**CKD-EPI**	Chronic Kidney Disease Epidemiology Collaboration
**CrCl**	Creatinine Clearance
**CVP**	Central Venous Pressure
**EGDT**	Early Goal Directed Therapy
**eGFR**	estimated Glomerular Filtration Rate
**ELWI**	Extravascular Lung Water Index
**EVLW**	Extravascular Lung Water
**GEDVI**	Global End-Diastolic Volume Index
**GFR**	Glomerular Filtration Rate
**HA**	Human Albumin
**HES**	Hydroxyethyl Starch
**HR**	Heart Rate
**ICU**	Intensive Care Unit
**IGFBP7**	Insulin-Like Growth Factor-Binding Protein 7
**IgG**	Immunglobulin G
**ITBVI**	Intrathoracic Blood Volume Index
**KDIGO**	Kidney Disease: Improving Global Outcome
**MAP**	Mean Arterial Pressure
**MDRD**	Modification of Diet in Renal Disease
**MPAP**	Mean Pulmonary Artery Pressure
**NAG**	β-N-Acetyl-β-D-Glucosaminidase
**NGAL**	Neutrophil Gelatinase-Associated Lipocalin
**NS**	Non-Significant
**PAOP**	Pulmonary Arterial Occlusion Pressure
**PCWP**	Pulmonary Capillary Wedge Pressure
**POD**	Postoperative Day
**PRBC**	Packed Red Blood Cell
**RAAS**	Renin-Angiotensin-Aldosterone System
**RAP**	Right Arterial Pressure
**RIFLE**	Risk, Injury, Failure, Loss, End-stage renal disease criteria for acute kidney injury
**RR**	Relative Risk
**RRT**	Renal Replacement Therapy
**SI**	Stroke Index
**SIRS**	Systemic Inflammatory Response Syndrome
**SOFA**	Sepsis-related Organ Failure Assessment
**SvO_2_**	Mixed Venous Oxygen Saturation
**SVR**	Systemic Vascular Resistance
**SVV**	Stroke Volume Variation
**TIMP2**	Tissue Inhibitor of Metalloproteinases 2
